# Experimentally validated full-vectorial model of wavelength multicasting via four-wave mixing in straight waveguides

**DOI:** 10.1038/s41598-018-31470-x

**Published:** 2018-08-29

**Authors:** Kai Guo, Jiacheng Feng, Xiaodong Shi, Jiehui Li, Minghong Gao, Hui Jing, Xiaolin Wang, Junbo Yang, Haiyan Ou

**Affiliations:** 1 College of Advanced Interdisciplinary Studies, National University of Defense Technology, Changsha, 410073 China; 2 Department of Photonics Engineering, Technical University of Denmark, Kgs. Lyngby, 2800 Denmark; 3 Center of Material Science, National University of Defense Technology, Changsha, 410073 China; 4 Shanghai Institute for Advanced Communication and Data Science, Key Laboratory of Information Science of Electromagnetic Waves, Fudan University, Shanghai, 200433 China; 5 College of Artificial Intelligence, National University of Defense Technology, Changsha, 410073 China

## Abstract

We derive full-vectorial nonlinear propagation equations of dual-pumped four-wave mixing in straight waveguides, which are valid in characterizing the one-to-six wavelength multicasting. Special attention is paid to the resulting idler wavelengths and their conversion efficiency, which enables the optimization of the experimental designs, including the incident wavelength and the power of pumps and signal. We validate the model by comparing the numerical simulation to the experimental measurement in a silicon-on-insulator waveguide, for the first time to our best knowledge, and achieve a good agreement. We further derive the general form of the proposed model for the case of using multiple,pumps, which holds a potential to numerically predict the performance of complex wavelength multicasting, and essentially guide the waveguide designs.

## Introduction

Four-wave mixing (FWM), a third-order nonlinear optical effect, makes possible a number of key features for all-optical signal processing^1^. Specifically, two pump photons (subscripted as *p* and *q*) annihilate and generate a pair of signal-idler photons (subscripted as *s* and *i*), in which the angular frequencies follow the energy conservation *ω*_*s*_ + *ω*_*i*_ = *ω*_*p*_ + *ω*_*q*_. FWM facilitates the energy conversion from one wavelength to another, known as the wavelength conversion^2^, or to several wavelengths, known as the wavelength multicasting^3^, which are of great significance in the wavelength division multiplexing networks, as the data packet is converted simultaneously^4^. While the initial experiments of FWM were carried out in highly nonlinear optical fibers^2,3^, the integrated platforms especially the silicon-on-insulator (SOI) waveguides, recently attract more research interest. SOI waveguides are compatible with the complementary mental-oxide semiconductor technology, which enables compact integration of opto-electronic components for building the on-chip optical communication system^5^. Moreover, SOI waveguides facilitate high refractive index contrast between the core and the cladding that ensures strong nonlinear interaction and tailorable group-velocity dispersion profile^6^ that benefits the phase matching^7^. Therefore, efficient wavelength conversion^8–13^ and wavelength multicasting^14–21^ have been demonstrated in a variety of waveguide designs, which takes the merits of broadband operation compared to that demonstrated in atomic or atomic-like mediums^22–24^.

However, few of the wavelength multicasting demonstrations pay attention to the numerical analysis. Previous studies^25^ have presented the nonlinear propagation equations in straight waveguides, yet by using the optical intensity with respect to the effective mode area, it remains difficult to directly compare the numerical simulations to the experimental measurements of the optical power. On the other hand, the theoretical models for optical fibers^26–28^, in which the scalar- approach is often assumed^29^, are invalid for straight waveguides because of the relatively large refractive index contrast. Although a good agreement can be achieved by fitting the parameters in the scalar-approach model, the numerical predictions may lead to mistakes when evaluating the nonlinear refractive index and optimizing designs of new waveguides. Hence, a full-vectorial model is of great importance, which facilitates a solid theoretical understanding, takes a simple form and can be easily validated by experiments.

In this paper, we derive the full-vectorial nonlinear propagation equations for dual-pumped FWM processes. We predict the idler wavelengths through the energy conservation and characterize the conversion efficiency of the one-to-six wavelength multicasting configuration. We validate the model by comparing the numerical simulations to the experimental measurements in a SOI waveguide sample and further give the general form of the model in the cases of using multiple pumps.

## Results

### Numerical model

The electric and magnetic fields are expanded in a set of continuous wave (CW) frequency components, *ω*_*n*_,

1 $$\{\begin{array}{c}{\bf{E}}({\bf{r}},t)\\ {\bf{H}}({\bf{r}},t)\end{array}\}=\frac{1}{2}\sum _{n}\,\frac{{A}_{n}(z)}{{N}_{n}}\{\begin{array}{c}{\bf{F}}(x,y)\\ {\bf{U}}(x,y)\end{array}\}\exp (\,-\,i{\omega }_{n}t+i{\beta }_{n}z)+{\rm{c}}{\rm{.}}\,{\rm{c}}{\rm{.}},$$

where **r** = {*x*, *y*, *z*} denotes the position coordinate, *A*_*n*_ denotes the common amplitude varying along the longitudinal direction *z*, **F**_*n*_ and **U**_*n*_ denote the field distribution functions of the electric and magnetic field in the transverse dimension, respectively, *β*_*n*_ denotes the propagation constant and c. c. denotes the complex conjugate. To ensure that |*A*_*n*_|^2^ equals the optical power *P*_*n*_ in watts, the normalization factor *N*_*n*_ is given by^30^

2 $${N}_{n}^{2}=\frac{1}{4}{\int }_{x,y}\,[{{\bf{F}}}_{n}\times {{\bf{U}}}_{n}^{\ast }+{{\bf{F}}}_{n}^{\ast }\times {{\bf{U}}}_{n}]\cdot {\bf{z}}\,dxdy,$$

where **z** denotes the unit vector of the longitudinal direction. *N*_*n*_ can take a simpler form

3 $$\begin{array}{l}{N}_{n}^{2}=\frac{c}{2{n}_{g}}{\int }_{x,y}\,{n}_{w}^{2}(x,y)|{{\bf{F}}}_{n}{|}^{2}\,dxdy,\end{array}$$

where *n*_*g*_ and *n*_*w*_(*x*, *y*) are the group index and the transverse refractive index distribution of the waveguide, respectively^31^. By substituting Eq. (1) into Maxwell’s equations, the propagation equation of the field amplitude at *ω*_*n*_ becomes^32–34^

4 $${\partial }_{z}{A}_{n}(z)=\frac{i{\omega }_{n}}{4{N}_{n}}{\int }_{S}\,{{\bf{F}}}_{n}^{\ast }\cdot {{\bf{P}}}_{n}^{NL}({\bf{r}})\,ds,$$

where ∂_*z*_ denotes the differentiation of *z*. Assuming that the electronic response is instantaneous, the Kerr-induced nonlinear polarization can be expanded with different frequency components,

5 $${{\bf{P}}}^{NL}({\bf{r}},t)=\frac{1}{2}\sum _{n}\,{{\bf{P}}}_{n}^{NL}({\bf{r}})\,\exp (\,-\,i{\omega }_{n}t+i{\beta }_{n}z)+{\rm{c}}{\rm{.}}\,{\rm{c}}\mathrm{.}$$

It can also be written as the tensor product

6 $${{\bf{P}}}^{NL}={\varepsilon }_{0}{\chi }^{\mathrm{(3)}}\vdots {\bf{EEE}},$$

where the third-order susceptibility tensor *χ*^(3)^ is assumed to be independent of frequency in the bandwidth of phase matching.

**Figure 1:**
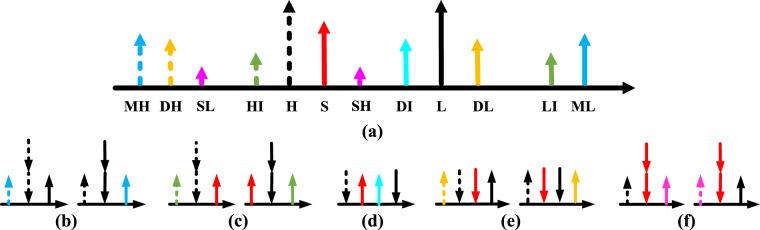
The output wavelengths and the corresponding energy conversion.

In the simple configuration we concerned, two pumps, subscripted as *H* (high frequency) and *L* (low frequency) and a signal, subscripted as *S*, are incident lights in the waveguide. The output wavelengths shown in Fig. 1 come from several FWM processes, which can be classified into the degenerate type pumped with the same frequency and the non-degenerate type pumped with different frequencies. Note that the incident wavelengths may behave as either the pump or the signal of FWM, but only the energy conversion involving *S* can be used for wavelength multicasting. Specifically, the degenerate FWM pumped at *H* generate the idler wavelengths of *MH* and *HI*, when the signal is at *L* and *S*, respectively. Similar processes pumped at *L* generate the idler wavelengths of *ML* and *LI*. Moreover, the non-degenerate FWM, using two of the incident lights as the pumps and the third one as the signal, generates the idler wavelengths of *DI*, *DH* and *DL*. Additionally, when the incident power of *S* is comparable to that of *H* and *L*, efficient degenerate FWM pumped at *S* may take place, that *H* and *L* as the signal generate the idler *SH* and *SL*, respectively.

Typically, all frequency components are in the same polarization state and within the bandwidth of phase matching, thus, the electric field distribution function **F** and the normalization factor *N* can be assumed to be frequency independent. Moreover, the spontaneous/cascaded/idler-induced processes bring negligible contributions to the output power at different wavelengths. To simplify the following descriptions particularly for the wavelength multicasting configuration, these terms are not concerned. Hence, assuming only ${\chi }_{0}^{\mathrm{(3)}}={\chi }_{iiii}^{\mathrm{(3)}}$ for *i* = *x* or *i* = *y* is nonzero, the nonlinearity induced polarization at *H* becomes

7 $${{\bf{P}}}_{{\rm{h}}}^{NL}=(|{A}_{{\rm{h}}}{|}^{2}+\mathrm{2|}{A}_{{\rm{l}}}{|}^{2}+\mathrm{2|}{A}_{{\rm{s}}}{|}^{2})\,{A}_{{\rm{h}}}\frac{{\varepsilon }_{0}}{4{N}^{3}}{\chi }_{0}^{\mathrm{(3)}}(\mathrm{2|}{\bf{F}}{|}^{2}{\bf{F}}+({\bf{F}}\cdot {\bf{F}}){{\bf{F}}}^{\ast }),$$

and by inserting Eq. (7) into Eq. (4), the propagation equation of *H* becomes

8 $${\partial }_{z}{A}_{{\rm{h}}}=i{\gamma }_{e}\,(|{A}_{{\rm{h}}}{|}^{2}+\mathrm{2|}{A}_{{\rm{l}}}{|}^{2}+\mathrm{2|}{A}_{{\rm{s}}}{|}^{2})\,{A}_{{\rm{h}}},$$

where the complex nonlinear coefficient is given by

9 $${\gamma }_{e}=\frac{\omega {\varepsilon }_{0}{n}_{g}^{2}}{4{c}^{2}}\frac{{\int }_{x,y}\,{\chi }_{0}^{\mathrm{(3)}}{{\bf{F}}}^{\ast }\cdot [\mathrm{2|}{\bf{F}}{|}^{2}{\bf{F}}+({\bf{F}}\cdot {\bf{F}}){{\bf{F}}}^{\ast }]dxdy}{{({\int }_{x,y}{n}_{w}^{2}|{\bf{F}}{|}^{2}dxdy)}^{2}}.$$

To have a convenient form known from optical fibers^29^, by only taking the nonlinear contribution of the core (footnoted by *c*) into account, the complex nonlinear coefficient can be written as

10 $${\gamma }_{e}=\frac{\omega {n}_{2}}{c{A}_{{\rm{eff}}}}+i\frac{{\beta }_{T}}{2{A}_{{\rm{eff}}}},$$

where the nonlinear refractive index and the two-photon absorption coefficient are identified as

11 $${n}_{2}=\frac{3{\rm{Re}}({\chi }_{c}^{\mathrm{(3)}})}{4{n}_{c}^{2}{\varepsilon }_{0}c},\,{\beta }_{T}=\frac{3\omega {\rm{I}}m({\chi }_{c}^{\mathrm{(3)}})}{2{n}_{c}^{2}{\varepsilon }_{0}{c}^{2}}.$$

In crystalline-semiconductor waveguides, the polarization coefficient *ζ*_*e*_ needs to be included in Eq. (11), which describes the nonlinear response with the polarization state. Finally, the resulting effective mode area, which is the key point of the full-vectorial model, is given by

12 $${A}_{{\rm{eff}}}=\frac{3}{{n}_{g}^{2}{n}_{c}^{2}}\frac{{({\int }_{x,y}{n}_{w}^{2}|F{|}^{2}dxdy)}^{2}}{{\int }_{c}{{\bf{F}}}^{\ast }\cdot [\mathrm{2|}{\bf{F}}{|}^{2}{\bf{F}}+({\bf{F}}\cdot {\bf{F}}){{\bf{F}}}^{\ast }]dxdy}\mathrm{.}$$

To account for loss mechanisms in straight waveguides, it is necessary to introduce the loss term in nonlinear propagation equations. Specifically in SOI waveguides, the wavelength-independent linear loss *α*_*l*_ and the wavelength-dependent free-carrier absorption^35,36^ are included in the loss coefficient given by

13 $${\alpha }_{n}={\alpha }_{l}+6\times {10}^{-10}\cdot \frac{{\lambda }_{n}^{2}\tau {\beta }_{T}\sum _{n}\,|{A}_{n}{|}^{4}}{\hslash \omega {A}_{{\rm{eff}}}^{2}},$$

where *τ* denotes the free carrier lifetime. At this point, the nonlinear propagation equation at *H* becomes

14 $${\partial }_{z}{A}_{{\rm{h}}}=-\,\frac{1}{2}{\alpha }_{{\rm{h}}}{A}_{{\rm{h}}}+i{\gamma }_{e}(|{A}_{{\rm{h}}}{|}^{2}+\mathrm{2|}{A}_{{\rm{l}}}{|}^{2}+\mathrm{2|}{A}_{{\rm{s}}}{|}^{2})\,{A}_{{\rm{h}}},$$

which can be transferred to that at *L* and *S* by swapping the footnote “h” with “l” and “s”, respectively. Moreover, at the degenerate FWM induced idler wavelengths such as *HI*, the nonlinear propagation equations need to involve the phase matching term as

15 $${\partial }_{z}{A}_{{\rm{hi}}}=-\,\frac{1}{2}{\alpha }_{{\rm{hi}}}{A}_{{\rm{hi}}}+i{\gamma }_{e}(\mathrm{2|}{A}_{{\rm{h}}}{|}^{2}+\mathrm{2|}{A}_{{\rm{l}}}{|}^{2}+\mathrm{2|}{A}_{{\rm{s}}}{|}^{2})\,{A}_{{\rm{hi}}}+i{\gamma }_{e}{A}_{{\rm{h}}}^{2}{A}_{{\rm{s}}}^{\ast }\,\exp [i\mathrm{(2}{\beta }_{{\rm{h}}}-{\beta }_{{\rm{s}}}-{\beta }_{{\rm{hi}}})z],$$

which can be transferred to that at *LI* by swapping the footnotes “hi” and “h” with “li” and “l”, respectively. Similarly, the nonlinear propagation equations at the non-degenerate FWM induced idler wavelengths such as *DI* is given by

16 $$\begin{array}{c}{\partial }_{z}{A}_{{\rm{di}}}=-\,\frac{1}{2}{\alpha }_{{\rm{di}}}{A}_{{\rm{di}}}+i{\gamma }_{e}(\mathrm{2|}{A}_{{\rm{h}}}{|}^{2}+\mathrm{2|}{A}_{{\rm{l}}}{|}^{2}+\mathrm{2|}{A}_{{\rm{s}}}{|}^{2}){A}_{{\rm{di}}}+i{\gamma }_{e}2{A}_{{\rm{h}}}{A}_{{\rm{l}}}{A}_{{\rm{s}}}^{\ast }\\ \,\,\,\times \exp [i({\beta }_{{\rm{h}}}+{\beta }_{{\rm{l}}}-{\beta }_{{\rm{s}}}-{\beta }_{{\rm{di}}})z],\end{array}$$

which can be transferred to that at *DH* or *DL*, by swapping the footnotes “di” and “s” with “dh” and “*l*” or with “dl” and “h”, respectively. As the data packet at *S* is efficiently converted into packets at five idler wavelengths (omitting *SL* and *SH*), which forms a one-to-six wavelength multicasting configuration, Eq. (14–16) and their equivalents, constituting the full-vectorial model, make possible the numerical predictions of the experimental results.

### Experimental validation

**Figure 2:**
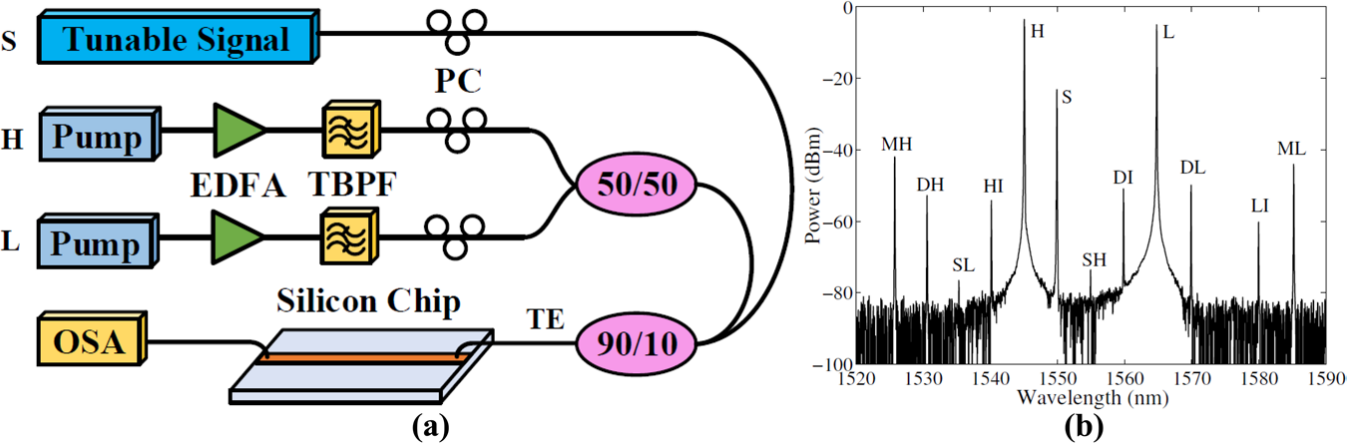
(**a**) A schematic of the validating experimental set up. EDFA: Erbium-doped fiber amplifier; TBPF: Tunable band-pass filter; PC: Polarization controller; OSA: Optical spectrum analyser. (**b**) The output spectrum when *H*, *L* and *S* are at 1545 nm, 1565 nm and 1550 nm, respectively.

We validate the derived full-vectorial model by comparing the numerical simulations of the conversion efficiency, given by the ratio of idler to signal power at the waveguide output, to the experimental measurements in a SOI sample. In the simulation, the nonlinear refractive index *n*_2_ is 6 × 10^−18^ m^2^/W, the two-photon absorption coefficient *β*_*T*_ is 4.5 × 10^−12^ m/W, the free carrier lifetime is 10 ns and the polarization coefficient *ζ*_*e*_ is 1.25 for the transverse electric (TE) mode^37^. The electric field profile is simulated through a finite difference mode solver^38^ and the resulting effective mode area at 1550 nm is estimated as 0.06 *μ*m^2^. Figure 2(a) shows the schematic of the validating experimental set up. Two pumps were power amplified by erbium-doped fiber amplifiers (EDFAs), where the incident amplified spontaneous emission was suppressed by tunable band-pass filters (TBPFs). The pumps were combined by a 50–50% coupler, followed by a 90–10% coupling with the tunable signal and were coupled in and out of the 1 cm waveguide sample through a pair of photonic crystal grating couplers (PCGCs)^39^. Polarization controllers (PCs) were used to control the polarization state, so that the coupling loss of PCGCs, which are designed for TE mode, reached the minimum (5 dB per facet). Finally, the output spectrum was measured by an optical spectrum analyser (OSA), shown in Fig. 2(b), where the generated wavelengths agree well with predictions.

**Figure 3:**
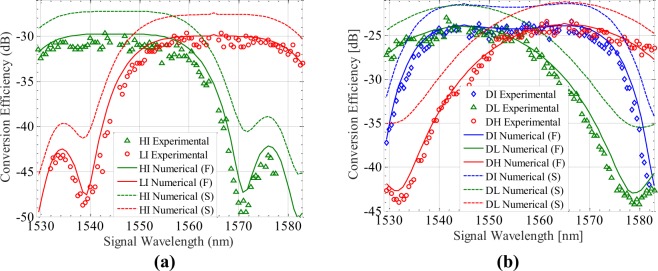
Full-vectorial numerical simulations (solid), Scalar-approach numerical simulations (dashed) and experimental measurements of the conversion efficiency at (**a**) *HI* (green triangles), *LI* (red circles), (**b**) *DI* (blue diamond), *DL* (green triangles) and *DH* (red circles). The incident power of pumps (1545 nm and 1565 nm) and signal are 10 dBm and −10 dBm, respectively.

We firstly set the pumps at 1545 nm and 1565 nm with the same in-waveguide incident power of 10 dBm. We simulate the conversion efficiency versus the signal wavelength using two effective mode area definitions and compare them to the experimental measurements with an incident signal power of −10 dBm. The experimental data, of which the idler power reaches the background noise level of the OSA, is omitted. Figure 3(a) shows that for the degenerate FWM induced idlers, *HI* and *LI*, the maximal conversion efficiency of −30 dB takes place when the wavelength of *S* approaches to that of *H* and *L*, respectively. Since the linear phase mismatch corresponding to the propagation constant difference of four waves in Eq. (15) remains negative, the conversion efficiency versus signal wavelength behaves top-flattened with a 1 dB bandwidth of 24 nm. Figure 3(b) shows that for the non-degenerate FWM induced idlers, the maximal conversion efficiency reaches −24 dB, due to the factor of 2 in the final term of Eq. (16) rather than the factor of 1 in the final term of Eq. (15). For *DL* and *DI*, such a maximum takes place when the wavelength of *S* approaches to that of *H*, while for *DH* and *DI*, such a maximum takes place when the wavelength of *S* approaches to that of *L*. The 1 dB bandwidth for *DI* reaches 34 nm, however for *DH* and *DL*, it reduces to only 16 nm because of the relatively larger linear phase mismatch depending on the pump detuning. Note that the effective mode area is estimated as 0.06 *μ*m^2^ and 0.04 *μ*m^2^, by using the full-vectorial definition and the scalar-approach definition, respectively. As can be seen, the full-vectorial simulations (solid) fit well with the experimental results, while the conversion efficiency predicted by the scalar-approach simulations (dashed) are about 3 dB higher. Such a comparison validates the accuracy of the proposed full-vectorial model, in addition to strengthening the results presented in the previous study^34^.

**Figure 4:**
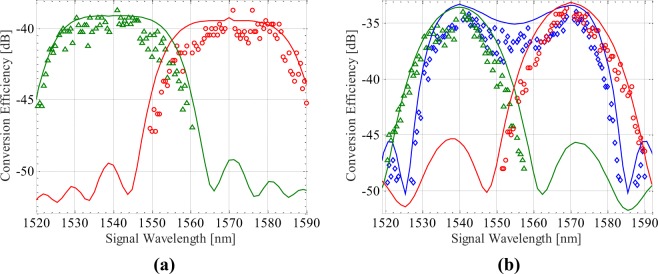
Numerical simulations (solid) and experimental measurements of the conversion efficiency at (**a**) *HI* (green triangles), *LI* (red circles), (**b**) *DI* (blue diamond), *DL* (green triangles) and *DH* (red circles). The incident power of pumps (1540 nm and 1570 nm) and signal are 5 dBm and −10 dBm, respectively.

We then set the pumps at 1540 nm and 1570 nm, meanwhile reduce the incident power downto 5 dBm. Figure 4(a) shows that for the degenerate FWM induced idlers, the maximal conversion efficiency reduces to −38 dB, but the 1 dB bandwidth remains unchanged, which reveals the fact that high incident pump power enables high conversion efficiency. Figure 4(b) shows that for the non-degenerate FWM induced idlers, the maximal conversion efficiency reduces to −33 dB. Although the pump detuning of 30 nm enables more separable idler wavelengths, the 1 dB bandwidth reduces to 24 nm for *DI* and 12 nm for *DH* and *DL*. The experimental measurements also agrees well with the numerical simulations, except for some small disagreement from the alignment drift, the incident power fluctuation and especially the unfiltered amplified spontaneous emission, which becomes more apparent by turning down the amplified power of EDFAs^40^.

Being experimentally validated, the proposed full-vectorial model makes possible the optimization of the experimental designs. The conversion efficiency becomes higher by turning up the incident pump power, but remains unchanged by turning up the incident signal power. The increase of the pump detuning may result in the reduction of the bandwidth that corresponds to efficient wavelength conversion. To achieve high conversion efficiency for all idlers, the signal wavelength needs to approach to but not equal to the central wavelength of the two pumps. Additionally, to broaden the bandwidth especially for the non-degenerate FWM induced idlers, the waveguide designs that enable near-zero anomalous group-velocity dispersion at concerned wavelengths are desired.

### General model for multiple-pumped wavelength multicasting

We derive the general form of the full-vectorial model for more complicated wavelength multicasting configuration using multiple pumps. Assume that *N* pumps and one signal are incident in the waveguide, where the initial power at all wavelengths are the same. The nonlinear propagation equation at one of the incident pumps, of which the amplitude is denoted by *A*_p_ for p = 1, 2, ... *N*, is given by

17 $${\partial }_{z}{A}_{{\rm{p}}}=-\,\frac{1}{2}{\alpha }_{{\rm{p}}}{A}_{{\rm{p}}}+i{\gamma }_{e}\,(\sum _{{\rm{p}}=1}^{N}\,\mathrm{2|}{A}_{{\rm{p}}}{|}^{2}-|{A}_{{\rm{p}}}{|}^{2}+\mathrm{2|}{A}_{{\rm{s}}}{|}^{2}){A}_{{\rm{p}}},$$

and can be transferred to that at the incident signal as

18 $${\partial }_{z}{A}_{{\rm{s}}}=-\,\frac{1}{2}{\alpha }_{{\rm{s}}}{A}_{{\rm{s}}}+i{\gamma }_{e}\,(\sum _{{\rm{j}}=1}^{N}\mathrm{2|}{A}_{{\rm{p}}}{|}^{2}+|{A}_{{\rm{s}}}{|}^{2}){A}_{{\rm{s}}}.$$

The degenerate FWM, governed by 2*ω*_*p*_ = *ω*_*s*_ + *ω*_*i*_, results in *N* idlers that follow

19 $${\partial }_{z}{A}_{{\rm{i}}}=-\,\frac{1}{2}{\alpha }_{{\rm{i}}}{A}_{{\rm{i}}}+i{\gamma }_{e}\,(\sum _{{\rm{p}}=1}^{N}\mathrm{2|}{A}_{{\rm{p}}}{|}^{2}+\mathrm{2|}{A}_{{\rm{s}}}{|}^{2}){A}_{{\rm{i}}}+i{\gamma }_{e}{A}_{{\rm{p}}}^{2}{A}_{{\rm{s}}}^{\ast \,}\exp [i\mathrm{(2}{\beta }_{{\rm{p}}}-{\beta }_{{\rm{s}}}-{\beta }_{{\rm{i}}})z].$$

The degenerate FWM, governed by 2*ω*_*s*_ = *ω*_*p*_ + *ω*_*j*_, also results in *N* idlers that follow

20 $${\partial }_{z}{A}_{{\rm{j}}}=-\,\frac{1}{2}{\alpha }_{{\rm{j}}}{A}_{{\rm{j}}}+i{\gamma }_{e}\,(\sum _{{\rm{p}}=1}^{N}\mathrm{2|}{A}_{{\rm{p}}}{|}^{2}+\mathrm{2|}{A}_{{\rm{s}}}{|}^{2}){A}_{{\rm{j}}}+i{\gamma }_{e}{A}_{{\rm{s}}}^{2}{A}_{{\rm{p}}}^{\ast }\,\exp [i\mathrm{(2}{\beta }_{{\rm{s}}}-{\beta }_{{\rm{p}}}-{\beta }_{{\rm{j}}})z].$$

Moreover, the non-degenerate FWM governed by *ω*_*p*_ + *ω*_*q*_ = *ω*_*s*_ + *ω*_*k*_, where *p* and *q* denote the subscripts of different incident pumps, results in *N*(*N*−1)/2 idlers that follow

21 $${\partial }_{z}{A}_{{\rm{k}}}=-\,\frac{1}{2}{\alpha }_{{\rm{k}}}{A}_{{\rm{k}}}+i{\gamma }_{e}(\sum _{{\rm{p}}=1}^{N}\,\mathrm{2|}{A}_{{\rm{p}}}{|}^{2}+\mathrm{2|}{A}_{{\rm{s}}}{|}^{2}){A}_{{\rm{k}}}+i{\gamma }_{e}2{A}_{{\rm{p}}}{A}_{{\rm{q}}}{A}_{{\rm{s}}}^{\ast }\,\exp [i({\beta }_{{\rm{p}}}+{\beta }_{{\rm{q}}}-{\beta }_{{\rm{s}}}-{\beta }_{{\rm{k}}})z].$$

Meanwhile, the non-degenerate FWM, governed by *ω*_*p*_ + *ω*_*s*_ = *ω*_*q*_ + *ω*_*m*_, results in *N*(*N*−1) idlers that follow

22 $${\partial }_{z}{A}_{{\rm{m}}}=-\,\frac{1}{2}{\alpha }_{{\rm{m}}}{A}_{{\rm{m}}}+i{\gamma }_{e}(\sum _{{\rm{p}}=1}^{N}\,\mathrm{2|}{A}_{{\rm{p}}}{|}^{2}+\mathrm{2|}{A}_{{\rm{s}}}{|}^{2}){A}_{{\rm{m}}}+i{\gamma }_{e}2{A}_{{\rm{p}}}{A}_{{\rm{s}}}{A}_{{\rm{q}}}^{\ast }\,\exp [i({\beta }_{{\rm{p}}}+{\beta }_{{\rm{s}}}-{\beta }_{{\rm{q}}}-{\beta }_{{\rm{m}}})z].$$

While the data packet at incident signal wavelength is not converted to other idler wavelengths, Eq. (17–22) constitute the full-vectorial model that characterizes the resulting wavelength multicasting configuration with the output wavelength amount of (3*N*^2^−*N* + 2)/2. Additionally, the general full-vectorial model is valid in other straight waveguides, by varying the loss coefficient, the complex nonlinear coefficient and noteworthy by using the effective mode area given by Eq. (12).

## Discussion

We derive the full-vectorial nonlinear propagation equations of the multiple-pumped four-wave mixing processes in straight waveguides, which takes the conventional simple form but becomes more accurate and study the dual-pumped case for one-to-six wavelength multicasting. We compare the numerical simulations of the conversion efficiency for the resulting idlers to the experimental measurements in a silicon-on-insulator waveguide sample and achieve a good agreement, which to our best knowledge is the first explicit validation of the numerical models. We characterize the conversion efficiency versus signal wavelength, which does not only show the incident signal wavelength, which produces high conversion efficiency for all idlers, but also enables the numerical prediction for the cases using multiple incident signals. We finally demonstrate the potential of the full-vectorial model to enlighten the experimental designs such as deciding pump/signal wavelength and the waveguide designs such as group-velocity dispersion tailoring, which benefit the future works of broadband wavelength multicasting configurations.

## Methods

### The SOI waveguide sample

The investigated waveguide sample is fabricated through the standard SOI nano-fabrication processes, including e-beam lithography and inductively coupled plasma etching. The height and the width of the waveguide are 250 nm and 450 nm, respectively. A silica cladding is deposited by the plasma enhanced chemical vapour deposition with a thickness of 1 *μ*m. The transmittance of the photonic crystal grating couplers centres at 1550 nm, with a 3 dB bandwidth of 29 nm. All concerned wavelengths have anomalous group-velocity dispersion, where the second-order derivation of the propagation constant with respect to the angular frequency, *β*_2_, is −1.4 ps^2^/m at 1555 nm.

### The model derivation and the numerical simulation

The key points of the full-vectorial model derivation are: introduce Eq. (3) to avoid the cross product calculation of electric and magnetic vectors; define Eq. (12) to ensure that the complex nonlinear coefficient of Eq. (10) takes the conventional form^25^ meanwhile the effective mode area becomes more accurate; predict all four-wave mixing processes to obtain the nonlinearity induced polarization contributed by all frequency components in Eq. (6); keep the terms being time-independent in the time integration of Eq. (4) and omit the terms contributed by generated idlers to simplify the final nonlinear propagation equations.

For a given waveguide structure, the cross-sectional distribution of the full-vectorial electric/magnetic fields and the resulting effective refractive index can be numerically calculated via open-source MATLAB codes^38^, from which we estimate the effective mode area and the propagation constant at all the concerned wavelengths, substitute them into Eq. (14–22) and calculate the propagation evolution of the field amplitude through the finite element method. Our MATLAB code for the conversion efficiency calculation can directly retrieve the field distribution and the effective refractive index from the open-source codes, so the wavelength-dependence and the full-vectorial property are well kept in our numerical simulations.

## Data Availability

The datasets generated and analyzed during the current study are available from the corresponding author on reasonable request.
